# The relationship between inspiratory lung function parameters and airway hyper-responsiveness in subjects with mild to moderate COPD

**DOI:** 10.1186/1756-0500-5-209

**Published:** 2012-04-30

**Authors:** Sunil K Ramlal, Frank J Visser, Wim C J Hop, Bas Staffhorst, P N Richard Dekhuijzen, Yvonne F Heijdra

**Affiliations:** 1 Dept. of Pulmonology, IJsselland Ziekenhuis, 2900 AR, Capelle a/d IJssel, 690, The Netherlands; 2 Dept. of Pulmonology, Canisius Wilhelmina Ziekenhuis, Nijmegen, The Netherlands; 3 Dept. of Biostatistics, Erasmus Medical Centre, Rotterdam, The Netherlands; 4 Dept. of Pulmonology, Radboud University Nijmegen Medical Centre, Nijmegen, The Netherlands

**Keywords:** Chronic obstructive pulmonary disease, Forced expiratory volume 1 second, Inspiratory lung functions parameters, Visual analogue scale

## Abstract

**Background:**

The aim of this study was to evaluate the effects of increasing doses of inhaled histamine on the forced expiratory volume in one second (FEV_1_), inspiratory lung function parameters (ILPs) and dyspnea in subjects with mild to moderate chronic obstructive pulmonary disease (COPD)

**Methods:**

Thirty-nine (27 males and 12 females) stable COPD patients (GOLD stages I and II) inhaled a maximum of six sequential doses of histamine according to ERS standards until one of these provocative doses produced a 20% decrease in FEV_1_ (PD_20_). The effects on the FEV_1_, the forced inspiratory volume in one second (FIV_1_), inspiratory capacity (IC), forced inspiratory flow at 50% of the vital capacity (FIF50), peak inspiratory flow (PIF) and dyspnea score by a visual analogue scale (VAS) were measured and investigated after each dose step

**Results:**

After each dose of histamine, declines in all of the lung function parameters were detected; the largest decrease was observed in the FEV_1_. At the PD_20_ endpoint, more FEV_1_ responders than ILP responders were found. Among the ILPs, the FIV_1_ and IC best predicted which patients would reach the PD_20_ endpoint. No significant correlations were found between any of the lung function parameters and the VAS results

**Conclusions:**

In COPD patients, the FEV_1_ and ILPs declined after each dose of inhaled histamine. FEV_1_ was more sensitive to histamine than the ILPs. Of the ILPs, FIV_1_ and IC were the best predictors of reaching the PD_20_ endpoint. No statistically significant correlations were found between the lung function parameters and the degree of dyspnea

## Background

Bronchial hyper-responsiveness (BHR) is defined as increased airway sensitivity to various inhaled pharmacological agents (e.g., histamine or methacholine) and physiological stimuli (e.g., cold air or hypotonic salt) resulting in narrowing of the bronchi. A BHR test is considered to be positive when a provocative concentration of less than 8 mg/ml of histamine or methacholine (PC_20_) or a provocative dose (PD_20_) of less than 7.8 μmol of these bronchoconstrictive agents causes a ≥20% decrease from the baseline value of the forced expiratory volume in one second (FEV_1_) [1]. There is an approximately 50% BHR prevalence rate in subjects with mild chronic obstructive pulmonary disease (COPD), and BHR is more prevalent in women than in men [2,3]. COPD patients with airway hyper-responsiveness have an elevated risk of experiencing an accelerated decline in FEV_1_[4,5].

Due to the structural changes and destruction that occur in the lung parenchyma in COPD patients, there is an increase in small airway resistance and a loss of lung elastic recoil, leading to a decline in the FEV_1_. It is assumed that these changes can lead to airflow obstruction [6,7]; however, the FEV_1_ appears to be a poor predictor of these changes [8]. Additionally, the correlation between FEV_1_ and dyspnea in COPD subjects is weak; a better correlation has been found between the forced inspiratory volume in one second (FIV_1_) and dyspnea [9].

Nevertheless, using FEV_1_ to evaluate the response to a bronchial challenge is widely accepted [10]. This acceptance is probably due to the simplicity of the measurement and its well-known reproducibility. Unlike FEV_1_ changes, the effects of inhaled histamine in COPD patients and the associated changes in inspiratory lung function parameters (ILPs) in stable, mild to moderate COPD patients are relatively unknown.

Dynamic airway compression during forced expiratory maneuvers can mask the effects of intervention with bronchodilators (e.g., salbutamol) or bronchoconstrictors (e.g., histamine). Because dynamic compression does not occur during inspiratory maneuvers, we reasoned that there should be more observable change in the ILPs than in the FEV_1_ after administering the bronchoconstrictor histamine. Therefore, we hypothesized that the ILPs may be more sensitive to histamine bronchoconstriction than the FEV_1_ and may correlate more closely with dyspnea.

The objectives of the present study included the following: to investigate the effects of inhaled histamine on the FIV_1_, inspiratory capacity (IC), forced inspiratory flow at 50% of the vital capacity (FIF50) and peak inspiratory flow (PIF), to study the changes in these inspiratory parameters at the time that the provocative dose produces a ≥20% decrease in the FEV_1_ and to investigate the correlation between the changes in these lung function parameters and the degree of dyspnea change, as measured by a visual analogue scale (VAS), when the bronchial challenge test is considered positive.

## Methods

### Study design

Forty patients (including 12 women) with stable COPD were recruited from the outpatient clinic of the pulmonary department of the Canisius Wilhelmina Hospital in Nijmegen. The subjects had COPD that was classified as mild to moderate (at GOLD (Global Initiative for Chronic Obstructive Lung Disease) stage I or II based on postbronchodilator FEV1 values [11,12]. The patients met the inclusion criteria if they had stable COPD, were between the ages of 40 and 80 years, were current or former smokers with at least a 10 pack-year history and did not demonstrate reversibility after using short-acting bronchodilators. Although reversibility can be defined as an increase in FEV1 that is both greater than 200 ml and 12% above the pre-bronchodilator FEV1 value, our patients were required to have historical records demonstrating less than 10% standardized reversibility (as a percentage of predicted FEV1) to short-acting bronchodilators [13,14].

All of the included patients were in a stable disease state. Five patients were being treated with short-acting beta-2-agonists, 4 patients with short-acting anticholinergics, 24 patients with long-acting beta-2-agonists, 19 patients with long-acting anticholinergics, 16 patients with inhaled corticosteroids and 2 patients with oral theophylline. No patients were using oral corticosteroids. The patients visited the spirometry laboratory in the morning after having refrained from using inhaled corticosteroids for at least one week, from using short-acting inhaled bronchodilators for at least 6 hours, from using long-term beta-2-agonist bronchodilators for 12 hours and from using tiotropium or theophylline for at least 24 hours. No patients received antihistamines during the week prior to the study. The patients were defined as clinically stable if they had not had COPD exacerbations or changes in their COPD medications over the previous 8 weeks, had not used oral corticosteroids for a period of two months prior to the study and had not used antibiotics within the previous month. The exclusion criteria included an FEV_1_ < 50% of the predicted value, oxygen therapy, being unable to complete the questionnaires and the presence of allergic rhinitis, asthma, heart disease, neuromuscular disorders and any known form of pulmonary malignancy.

The Medical Ethics Committee of Arnhem-Nijmegen in the Netherlands gave permission for this study, and all of the patients gave their written informed consent prior to study participation.

### The histamine provocation test

The histamine inhalation tests were performed according to the ERS standards [1]. The histamine solutions were stored at 4 degrees Celsius and were administered at room temperature. Six doses of histamine-acetyl-beta-methylcholine-chloride (0.04 mg, 0.09 mg, 0.18 mg, 0.29 mg, 0.71 mg and 1.30 mg, yielding a cumulative dose ranging from 0.04 to 2.60 mg) in normal saline were inhaled by tidal breathing for 1 min at 5-min intervals using a Koko DigiDoser dosimeter (PPS Research, Louisville, CO, USA). BHR was defined as a PD_20_ < 2.60 mg (8.48 μmol). The PD_20_ value is the provocative dose of inhaled histamine that produces a decrease of 20% or more in the FEV_1_.

The baseline standard spirometry tests were performed first. Five FIV_1_ maneuvers were performed according to the procedures reported in our previous study, in which the subjects were asked to exhale slowly until reaching the residual volume level and to subsequently perform a forced, deep inspiration until reaching the total lung capacity (TLC) level [15]. The best values for the FIV_1_, IC, FIF_50_ and PIF were obtained from these data. Subsequently, three FEV_1_ flow volume curves were obtained according to the ERS standards, and the best FEV_1_ was recorded. The IC was measured immediately before each forced inhalation using the method described by Hadcroft and Calverly [16]. If the vital capacity (VC) was reached before the FIV_1_ during the inspiratory maneuvers, then it was assumed that FIV_1_ = VC. The largest FVC and FEV_1_ values were recorded. The reference values from the European Community for Steel and Coal were used for the predicted FEV_1_ and forced vital capacity (FVC) values [17]. In the current study, the subjects who were exhausted and whose FIV_1_ and FEV_1_ values decreased by less than 10% from the baseline values were restricted to three forced inspiratory and three forced expiratory maneuvers. For the rest of the subjects, five forced inspiratory and three forced expiratory maneuvers were performed. After a pre-test with a saline aerosol solution, the challenges with sequential inhalations of the histamine aerosol began. A 1-min rest interval was provided between each dose of histamine. Afterwards, the above-mentioned standard lung function tests were performed. The challenge was terminated when there was an FEV_1_ decline of at least 20% or after the maximum histamine dose was administered. The patients with more than a 20% decrease in the FEV_1_ value after histamine administration were treated with four puffs of fenoterol 100 mcg/ipratropium 20 mcg to aid their recovery. For each patient, the PD_20_ value of the inhaled histamine was estimated by log-linear interpolation [18]. We used the one-hour repeatability of these lung function parameters (the random variation expressed as the coefficients of repeatability (CR)) to compare the effects of the inhaled histamine on the ILPs and FEV_1_[19].

### Dyspnea score

The patients rated changes in dyspnea post-histamine administration using a VAS scale. The VAS scale is a 10-cm-long horizontal line that ranges from −5 to + 5 cm. VAS = −5 indicates significantly improved dyspnea, VAS = 0 indicates no change, and VAS = +5 indicates significantly worsened dyspnea. Prior to the test, all subjects were instructed on how to use the VAS scale.

### Statistical analysis

The one-sample *t*-test was used to compare the mean changes in lung function parameters from the baseline to the changes following different histamine dose steps. A repeated measures analysis of variance, ANOVA, was used to compare the mean changes in the various lung function parameters at the different dose steps. Pairwise comparisons of the lung function changes were made using the paired *t*-test. Spearman’s correlation coefficients (r_S_) were calculated. Receiver Operating Characteristic (ROC) curves was constructed to investigate the predictive value of the percentage change from baseline in the various ILPs in regard to reaching the PD_20_ endpoint. The areas under the curve (AUC) were calculated, and an AUC > 0.80 was considered to denote good predictive value. The analyses were performed using SPSS version 15.1 for Windows. A difference with a two-sided p-value < 0.05 was considered to be significant. Among the 39 included patients, we expected that at least 36 would reach the PD_20_ endpoint. With 36 patients, a correlation of 0.45 or greater between the FEV_1_ or ILP changes from baseline and the VAS could be detected at an alpha of 0.05 with a power of at least 80%.

## Results

### Patient characteristics

The baseline lung function values and patient characteristics are listed in Table 1. Only one of the forty subjects was excluded from the study. This subject developed persistent, severe dyspnea and had an FEV_1_ decrease of almost 19% after the first dose of histamine. One of the subjects stopped participating after the fourth dose; therefore, only seven patients participated up to dose step 5. Furthermore, two patients did not exhibit a significant response, despite being administered the highest dose.

**Table 1:** The patient characteristics and baseline lung function parameters for the 39 subjects in this study

Male/Female ratio, numbers (percentage)	12/27 (31%/69%)
Age, years	66 ± 7
Smoker, (ex-/current)^$^	13/26 (33%/67%)
GOLD stage 1 patients, number	10
GOLD stage 2 patients, number	29
FEV_1_, L/s (pre-bronchoconstriction)	1.89 ± 0.46
FVC, L (pre-bronchoconstriction)	3.11 ± 0.72
FEV_1_/FVC (%, pre-bronchoconstriction)	61.1 ± 8.1
FEV_1_ (% predicted)	67.0 ± 11.1
FEV_1,_ L/s (post-bronchoconstriction)	1.67 ± 0.49
FVC, L (post-bronchoconstriction)	2.83 ± 0.73
FIV_1_, L/s	2.92 ± 0.75
IC, L	2.32 ± 0.53
FIF_50_, L/s	4.97 ± 1.40
PIF, L/s	5.40 ± 1.48
PD_20_, mg	0.13 (0.04 to >2.60^#^)

### Changes in lung function parameters after each dose of histamine during the histamine provocative test

**Figure 1:**
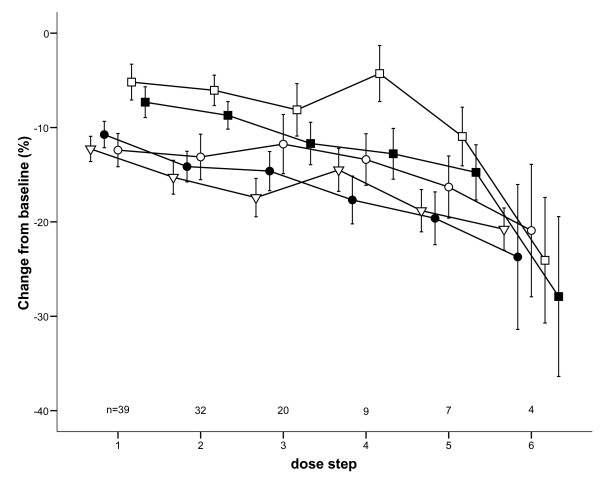
**The changes from the baseline values (± SEM) of the FIF**_**50**_**(open squares), PIF (solid squares), IC (open circles), FIV**_**1**_**(solid circles) and FEV**_**1**_**(triangles) after each dose of histamine;n = number of subjects tested at each histamine dose step.**

The effect of histamine on the various ILPs is shown in Table 2. Following the last administered dose of histamine, two of the four subjects did not display a positive provocation response. Figure 1 presents the mean changes from the baseline values observed at the various dose steps. All of the mean changes up to the sixth dose step were significantly less than zero, with the exception of the FIF_50_ at the fourth dose step. The ANOVA indicated significant differences between the various lung function changes at dose steps 1, 2 and 3 (all *p* < 0.006). The FEV_1_ generally displayed the largest decrease at these dose steps, and the FIF_50_ displayed the smallest. At dose steps 1, 2 and 3, the mean FEV_1_ decrease was significantly greater than the corresponding FIF_50_ and PIF changes. No significant differences were found between the FEV_1_ changes and the changes in the FIV_1_ or IC. Possibly due to the small number of remaining patients, no significant differences were found among the measured parameters at dose steps 4, 5 or 6.

**Table 2:** The percentage changes from the baseline lung function parameters and VAS dyspnea scores after each of the six histamine doses

**Mean values(± SD)**						
	dose step 1	dose step 2	dose step 3	dose step 4	dose step 5	dose step 6
FEV_1_	-12.0 ± 7.8	-15.2 ± 10.1	-17.4 ± 9.1	-14.4 ± 6.8	-18.8 ± 5.9	-20.7 ± 4.5
FIV_1_	-10.4 ± 8.4	-14.1 ± 9.2	-14.6 ± 9.2	-17.6 ± 7.6	-19.6 ±7.4	-23.7 ± 15.3
IC	-12.4 ± 10.5	-13.1 ± 13.6	-11.7 ± 14	-13.3 ± 8.2	-16.3 ± 8.7	-20.9 ± 14.0
FIF_50_	-5.2 ± 11.3	-6.0 ± 9.1	-8.1 ± 12.4	-4.2 ± 8.9	-10.9 ± 8.2	-24.0 ± 13.3
PIF	-7.3 ± 9.7	-8.7 ± 8.2	-11.6 ± 10	-12.7 ± 8.1	-14.7 ± 7.7	-27.9 ± 16.9
VAS	0.3 ± 1.0	0.7 ± 1.1	1.3 ± 0.9	1.3 ± 1.0	1.6 ± 1.2	1.2 ± 1.6
N1	39	32	20	9	7	4
N2	7	12	11	1	3	2

Definition of abbreviations:

FEV_1_ = forced expiratory volume in 1 second

FIV_1_ = forced inspiratory volume in 1 second

IC = inspiratory capacity (L)

FIF_50_ = forced inspiratory flow at 50% of the vital capacity (L/s)

PIF = peak inspiratory flow (L/s)

VAS = VAS score for the change of dyspnea after each histamine dose

N1 = number of subjects tested at the given histamine dose

N2 = number of subjects with a FEV_1_ decrease from baseline of ≥ 20% at the given histamine dose.

### Changes in lung function parameters after inhaling histamine when PD_20_ is reached

**Figure 2:**
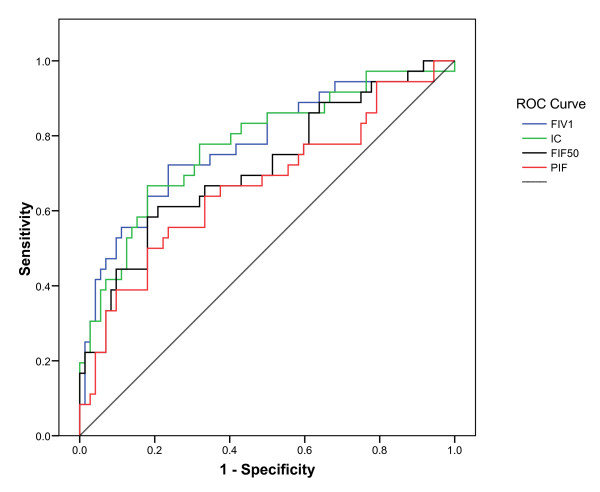
**The ROC curves for predicting the occurrence of an FEV**_**1**_**decrease ≥20% using the changes from the baseline values (%) of the FIV**_**1**_**, IC, FIF**_**50**_**and PIF.** The areas under the curve are 0.78, 0.78, 0.71 and 0.66. The dotted line represents a test with no predictive value.

The PD_20_ endpoint was reached and the one-hour CR was investigated in 36 of the 39 patients. We defined a responder as a subject who demonstrated a change in a parameter value greater than the following CRs: 12% for FEV_1_, 14% for FIV_1_, 19% for IC, 21% for FIF_50_ and 18% for PIF [19]. The results are summarized in Table 3. At PD_20_, more FEV_1_ responders were found than ILP responders. FIV_1_ and the IC were more sensitive than the flow parameters (FIF_50_ and PIF). To investigate which ILP was the best predictor of the occurrence of the PD_20_ endpoint, ROC curves were constructed for each ILP. Figure 2 demonstrates that the changes from the baseline FIV_1_ and IC values had the best predictive values (both AUCs were equal to 0.78). The predictive values of the FIF_50_ and PIF were somewhat lower, with AUCs of 0.71 and 0.67, respectively.

**Table 3:** **The ILP changes upon reaching the PD**_**20**_**endpoint in 36 patients, stratified by the coefficient of repeatability of the various lung function parameters**

**response criterion**	**non-responder/responder (at PD_20_ endpoint FEV_1_)**
FEV_1_ response >12%*	0 vs. 36 (0% vs. 100%)
FIV_1_ response >14%*	10 vs. 26 (28% vs. 72%)
IC response > 19%*	14 vs. 22 (39% vs. 61%)
FIF_50_ response >21%*	28 vs. 8 (78% vs. 22%)
PIF response >18%*	24 vs. 12 (67% vs. 33%)

Definition of abbreviations:

* = one-hour coefficient of repeatability of the particular lung function parameter

FEV_1_ = forced expiratory volume in 1 second

FIV_1_ = forced inspiratory volume in 1 second

IC = inspiratory capacity (L)

FIF_50_ = forced inspiratory flow at 50% of the vital capacity (L/s)

PIF = peak inspiratory flow (L/s)

The relationships between the percentage changes from the initial FEV_1_ values and the changes in the ILPs after the PD_20_ endpoint dose was reached are presented in Table 4. The mean percentage changes for all of the ILPs differed significantly from the corresponding changes in FEV_1_ (*p* < 0.04 for all parameters). In addition, all of the mean changes in the ILPs differed significantly from each other, with the exception of the changes in FIV_1_ versus the changes in IC (*p* = 0.75). Only the changes in FIV_1_ correlated significantly with the changes in FEV_1_. After reaching the PD_20_ endpoint, 8 of the 36 subjects had a decrease of more than 20% in their FIF_50_ and PIF values.

**Table 4:** **The mean percentage changes from the initial FEV**_**1**_**and ILP values in 36 subjects after inhaling histamine at the endpoint dose step (FEV**_**1**_**decrease ≥20%)**

	**mean percentage changes from initial values ± SD**	**correlation (r_S_) with changes in FEV_1_**
FEV_1_	-24.9 ± 3.7 (n.t.)	-
FIV_1_	-20.1 ± 9.3 (p < 0.001)	0.47(p = 0.004)
IC	-20.6 ± 12.5 (p < 0.001)	0.22 (p = 0.206)
FIF_50_	-12.6 ± 11.1 (p < 0.001)	0.17 (p = 0.322)
PIF	-14.6 ± 11.2 (p < 0.001)	0.24 (p = 0.161)

Definition of abbreviations:

FEV_1_ = forced expiratory volume (L) in 1 second

FIV_1_ = forced inspiratory volume (L) in 1 second

IC = inspiratory capacity (L)

FIF_50_ = forced inspiratory flow at 50% of the vital capacity (L/s)

PIF = peak inspiratory flow (L/s)

n.t. = not tested (less than -20% by design)

### Relationship between changes in lung function parameters and changes in dyspnea measured with the VAS

The mean VAS score gradually increased with increasing histamine doses, indicating an increase in dyspnea from dose step 1 to dose step 5 (*p* < 0.001). No significant correlations were found between the changes in the lung function parameters and the VAS score at the dose where the FEV_1_ had fallen by 20% or more from the baseline (Table 5).

**Table 5:** **The Spearman correlations of the VAS results with the changes (%) from the initial lung function values in 36 subjects after inhaling histamine at the endpoint dose step (FEV**_**1**_**decrease ≥20%)**

	**∆ FEV_1_- VAS**	**∆FIV_1_- VAS**	**∆IC-**	**∆ FIF_50_- VAS**	**∆PIF-VAS**
r_S_	0.12	-0.10	0.08	-0.33	-0.30
p-value	0.482	0.554	0.655	0.053	0.079

Definition of abbreviations:

FEV_1_ = forced expiratory volume in 1 second

FIV_1_ = forced inspiratory volume in 1 second

IC = inspiratory capacity (L)

FIF_50_ = forced inspiratory flow at 50% of the vital capacity (L/s)

PIF = peak inspiratory flow (L/s)

∆ = changes in initial values of lung function parameters when reaching the PD_20_ endpoint

A higher VAS score denotes a higher degree of dyspnea

## Discussion

The results of the present study demonstrate that the ILPs and FEV_1_ are sensitive to broncho-obstructive challenges. After each dose of histamine, there was a statistically significant decline in the FEV_1_ and ILPs, with the largest decrease being observed in the FEV_1_. The declines among the ILPs differed. The changes in the IC and FIV_1_ were larger than the FIF_50_ and PIF responses. Until now, no studies have examined the effects of histamine on the ILPs of subjects with mild to moderate forms of COPD. A greater number of FEV_1_ responders than ILP responders were found. Furthermore, the nadir was more pronounced in the FEV_1_ group than in the ILP group. Because the FEV_1_ changes were more sensitive to inhaled histamines compared to the ILP changes, we rejected our hypothesis that the ILPs might be more sensitive tools for detecting a bronchoconstrictive response than the FEV_1_. This finding may have been due to the small number of patients who participated in this study, but this result is consistent with a previous finding that FEV_1_ is a more sensitive parameter for detecting a bronchodilator response than the ILPs [20].

Of all of the ILPs, only changes in FIV_1_ were found to have a significant (but moderate) correlation with the corresponding changes in FEV_1_ (r_S_ = 0.47, *p* = 0.004) at the PD_20_ endpoint. Only 8 of the 36 subjects had a decrease of more than 20% in FIF_50_ and PIF. Additionally, the FIV_1_ and IC were more sensitive than the FIF_50_ and PIF. The FIV_1_ and IC changes from baseline again had the best predictive value for determining the patients who would reach the endpoint. There are currently no reports in the literature that support this finding. An overview of our PD_20_ endpoint data indicates that only two of the included subjects did not have a positive challenge test at the final dose. The majority of the population (30 subjects or approximately 77%) demonstrated a positive challenge test within the first three histamine doses. In a study by Kanner et al., the prevalence of BHR in subjects with mild COPD varied between 25-48%; furthermore, the prevalence of BHR was reported to be approximately 66% in mild or early COPD subjects in the Lung Health Study [21,22]. However, a study by Taube et al. of nine subjects with stable, mild to severe COPD found a positive challenge test for all nine subjects after administering inhaled histamine [23]. Asthma patients were excluded from our study. We made an active effort to exclude patients with diagnosed or possible undiagnosed asthma with well-defined inclusion and exclusion criteria.

The high histamine sensitivity of our study population may have been due to several reasons. First, 13 subjects (33%) were current smokers, and 26 subjects (67%) were former smokers. In smokers with mild COPD, the presence of many mast cells in the COPD airways may lead to BHR [24]. Second, some of these subjects had gastro-esophageal reflux, which can induce BHR [25]. Third, some of the subjects had used inhaled corticosteroids (ICS). Withdrawal of ICS, which are known to reduce the maximal degree of airway narrowing, prior to entering the study may have led to an increased sensitivity to bronchoconstriction. While ICS have been proven to be important in reducing BHR in asthmatic subjects, they have not been found to be particularly effective in subjects with COPD [26]. Jarad et al. suggested that even in subjects with COPD, the abrupt withdrawal of inhaled corticosteroids should be instituted carefully [27].

No statistically significant correlations were found between the lung function parameters and the degree of dyspnea as measured by the VAS. These findings are inconsistent with those of Taube et al., who found a more significant correlation between the relative FIV_1_ changes (r = 0.730, *p* < 0.001) and VAS than between the FEV_1_ changes (r = 0.389, *p* < 0.01) and VAS after administering the bronchodilator salbutamol in patients with stable COPD [28]. The finding that FEV_1_ and ILPs do not correlate with dyspnea is consistent with the results of our previously mentioned study of 85 stable COPD patients, in which we found no significant VAS changes after using short-term bronchodilators. Therefore, we also rejected our hypothesis that ILPs correlated more strongly with dyspnea following a histamine challenge. This difference may also have been due to the small number of patients who participated in our study and to our patient population having mild to moderate COPD rather than severe COPD, as in the study by Taube. Another possible explanation for the poor correlation between the FEV_1_ and VAS in our study is that most of the subjects had stable COPD for at least two months and were probably low perceivers [29]. The most common lung function variables used to evaluate the degree of correlation with dyspnea in COPD subjects are the PEF and FEV_1_[30]. There are no other available studies of the sensitivity of dyspnea scores in COPD subjects that have investigated the perception of dyspnea after administering inhaled histamine.

## Conclusions

We found that the FEV_1_ appeared to be more sensitive than the ILPs to increasing doses of inhaled histamine in subjects with stable, mild to moderate COPD. Although there was a decline in the FEV_1_ and in all of the ILPs, the largest decrease observed was in the FEV_1_. In vition, a greater number of responders were found when we focused on the FEV_1_ rather than on the ILPs. The FIF_50_ and the PIF appeared to be the least sensitive measures. Of the ILPs, the FIV_1_ and IC were the best predictors of which patients would reach the PD_20_ endpoint. No statistically significant correlations were found between the lung function parameters and the degree of dyspnea as measured by the VAS.

## Abbreviations

BHR, Bronchial hyper-responsiveness; FEV1, Forced expiratory volume in 1 s; FIV1, Forced inspiratory volume in 1 s; IC, Inspiratory capacity (L); FIF50, Forced inspiratory flow at 50% of the vital capacity (L/s); PIF, Peak inspiratory flow (L/s); PD20, Dose of histamine causing ≥ 20% decrease in FEV1 from the baseline value; VAS, Visual analogue scale; GOLD, Global initiative for chronic obstructive lung disease; ILPs, Inspiratory lung function parameters.

## Competing interest

The authors declare that they have no competing interests.

## Authors’ contributions

SKR managed the project and was responsible for the patients in the study. FV coordinated the study. BS carried out initial data. WH performed the statistical analysis.PRD and YH had the idea for the study.SKR, FV, PRD and YH all contributed to the writing of the paper. All authors read and approved the final manuscript.
